# Sound Categories: Category Formation and Evidence-Based Taxonomies

**DOI:** 10.3389/fpsyg.2018.01277

**Published:** 2018-07-30

**Authors:** Oliver Bones, Trevor J. Cox, William J. Davies

**Affiliations:** Acoustics Research Centre, University of Salford, Salford, United Kingdom

**Keywords:** soundscape, everyday sounds, taxonomy, categories, category formation, valence, arousal, acoustic correlates

## Abstract

Five evidence-based taxonomies of everyday sounds frequently reported in the soundscape literature have been generated. An online sorting and category-labeling method that elicits rather than prescribes descriptive words was used. A total of *N* = 242 participants took part. The main categories of the soundscape taxonomy were people, nature, and manmade, with each dividing into further categories. Sounds within the nature and manmade categories, and two further individual sound sources, dogs, and engines, were explored further by repeating the procedure using multiple exemplars. By generating multidimensional spaces containing both sounds and the spontaneously generated descriptive words the procedure allows for the interpretation of the psychological dimensions along which sounds are organized. This reveals how category formation is based upon different cues – sound source-event identification, subjective-states, and explicit assessment of the acoustic signal – in different contexts. At higher levels of the taxonomy the majority of words described sound source-events. In contrast, when categorizing dog sounds a greater proportion of the words described subjective-states, and valence and arousal scores of these words correlated with their coordinates along the first two dimensions of the data. This is consistent with valence and arousal judgments being the primary categorization strategy used for dog sounds. In contrast, when categorizing engine sounds a greater proportion of the words explicitly described the acoustic signal. The coordinates of sounds along the first two dimensions were found to correlate with fluctuation strength and sharpness, consistent with explicit assessment of acoustic signal features underlying category formation for engine sounds. By eliciting descriptive words the method makes explicit the subjective meaning of these judgments based upon valence and arousal and acoustic properties, and the results demonstrate distinct strategies being spontaneously used to categorize different types of sounds.

## Introduction

Categorization is a fundamental process by which meaning is applied to sensory experience (Dubois, 2000) based upon the correlational structure of the attributes of objects in the environment (Rosch, 1978). Knowledge about the environment is parsed and organized according to category structures. This simplifies the environment and gleans information with less cognitive effort (“cognitive economy"; Rosch, 1978), with inferences assuming that category members have similar attributes.

Which attributes of the many sounds experienced in everyday life are used to form categories? One approach to answering this question is the semantic differential method, whereby participants score concepts and events on a number of attribute rating scales. Typically this is followed by factor analysis in order to extract the principal dimensions which are then interpreted according to the attribute scales with which they most strongly correlate. Classically the results from this type of method are said to demonstrate that the factors ‘evaluation,’ ‘potency,’ and ‘activity’ (EPA) characterize the affective components of meaning (Osgood, 1952, 1969), and that this occurs universally across cultures (e.g., Heise, 2001). A number of studies have used this method for soundscapes, finding dimensions analogous to EPA, such as ‘pleasantness’ (Björk, 1985; Payne et al., 2007; Axelsson et al., 2010; Hong and Jeon, 2015), ‘preference’ (Kawai et al., 2004; Yu et al., 2016), ‘calmness’ (Cain et al., 2013), ‘relaxation’ (Kang and Zhang, 2010), ‘dynamic’ (Kang and Zhang, 2010), ‘vibrancy’ (Cain et al., 2013), ‘playfulness’ (Yu et al., 2016), and ‘eventfulness’ (Axelsson et al., 2010; Hong and Jeon, 2015). A number of other, possibly more sound-specific components are also reported, such as ‘sense of daily life’ (Kawai et al., 2004), ‘familiarity’ (Axelsson et al., 2010), ‘spatiality’ (Kang and Zhang, 2010), ‘harmony’ (Hong and Jeon, 2015), ‘communication’ (Kang and Zhang, 2010), ‘loudness,’ and ‘richness’ (Yu et al., 2016). A related framework is that of ‘core affect’ (for a review see Russell, 2003); this is a dimensional model of affective states as the linear combination of valence (a pleasure–displeasure continuum) and arousal (an alertness continuum).

Cluster analysis of semantic differential data identifies groups of sounds that are considered similar in terms of the attribute scales used, and factor analysis identifies the underlying dimensions of ratings on those scales. As these attributes are prescribed *a priori* by the experimenter, however, the dimensions may lack ecological validity for understanding categorization. An alternative approach is to generate similarity date by pairwise comparison (e.g., Gygi et al., 2007) or by sorting tasks. This approach avoids prescribing attributes on which to rate sounds, although in the case of pairwise comparisons the time required to perform comparisons can be prohibitively long. Moreover, since the procedure does not generate semantic labeling the meaning of the resulting categories must be interpreted by the researcher. Sorting tasks on the other hand produce similarity data which can be interpreted by linguistic analysis of the category descriptions. Since this approach allows participants to form categories using their own criteria and to provide their own descriptors, this method provides insight into how categories are formed with greater ecological validity than the semantic differential method.

Dubois et al. (2006) used a sorting method to investigate soundscapes consisting of sounds containing human activity. The results produced categories formed principally by similarity of sound sources and places. For categories consisting of sounds that were identified as containing noises, these were categorized by similar sources or actions. Another study by Guastavino (2007) asked participants to sort ambient urban noise. Similar to Dubois et al. (2006), categories were principally differentiated by those that contained sounds consisting of mostly human activity or those that contained sounds consisting mostly of traffic noise. Subcategories were formed around type of activity. Likewise, Morel et al. (2012) found that categories of road traffic noise were formed based upon vehicle type (sound source) and driving condition (action).

These categorizations and dimensions relate to complex environmental sounds, and are consistent with Guastavino (2006). In this study a linguistic analysis of interview data found that descriptions of sound sources accounted for 76% of the descriptions of the soundscapes. With respect to detached sounds, using a similar sorting and labeling procedure Houix et al. (2012) identified categories of domestic noises based on temporal extent, which resembled those previously proposed by Gaver (1993), based upon the type of material (e.g., solid) and events (e.g., impact) producing the sound. Previous work using semantic differentials has identified dimensions, such as ‘identifiability,’ ‘timbre,’ and ‘oddity’ (Ballas, 1993), EPA (Björk, 1985), and ‘harshness,’ ‘complexity,’ ‘appeal,’ and ‘size’ (Kidd and Watson, 2003). As noted above, these are not necessarily an ecologically valid representation of the criteria by which categories are formed. Using a hierarchical sorting paradigm, Giordano et al. (2010) found evidence for symbolic (acoustic) properties predicting similarity of environmental sounds from non-living objects, whereas iconic (semantic) meaning predicted similarity of environmental sounds from living things (see also results of neuroimaging studies by, e.g., Lewis et al., 2005). Finally, a recent study by Bergman et al. (2016) found evidence for valence and arousal contributing to the first dimension of data from a pairwise dissimilarity rating task with everyday sounds, suggesting that emotional response may also play a role in categorization.

Our study explored the formation of categories for a set of everyday sounds that are frequently reported in the soundscape literature. Evidence-based taxonomies were developed in order to explore the formation of categories at different levels of hierarchy. In order to test the hypothesis that the use of cues for category formation would differ both between levels of the emergent taxonomy and between different sounds within levels, we performed a statistical analysis of verbal correlates of sound category formation. The different ways that people use cues to form sound categories have important implications for research in everyday sound. The relationship between sound category formation and emergent sound taxonomies sheds light on the perception of everyday sound.

## Materials and Methods

### Procedure

Multiple measurements revealed the formation of categories at different levels of the emergent taxonomy. The top level experiment tested ‘soundscape,’ the middle ‘nature’ and ‘manmade,’ and the bottom ‘dogs’ and ‘engines.’ The categories formed at the ‘top’ level of the taxonomy informed the selection of sounds for studies at the ‘middle’ level, and individual sound sources from the ‘middle’ level were selected for a study of ‘bottom’ level sounds. Each study was conducted via a web interface on Sound101^1^, a website hosted by one of the authors. Each sound was represented by a tile containing a single word descriptor (e.g., ‘Road_1’), all of which were arranged in a random order in a ‘sound bank’ panel on the left hand side of the screen at the onset of the study. In the case of the dogs and engines studies, tiles were labeled as ‘Dog_1,’ ‘Dog_2’ etc. Instructions at the top of the screen directed participants to: click the tiles to hear the sound; group similar sounds together by dragging them from the sound bank into one of five categories; use all five categories; give each category a name describing the sounds in the category. In addition, participants were instructed not to use category names, such as ‘miscellaneous,’ ‘random,’ or ‘sounds’ etc. A pilot study found that five was the mean number of categories used when freely sorting the 60 sounds from the top level study. No time limit was imposed, and the average time taken was approximately 20 min. The procedure was approved by the University of Salford Science & Technology Research, Innovation and Academic Engagement Ethical Approval Panel.

### Stimuli and Participants

All participants completed a short web form prior to the experiment consisting of questions on age, sex, and audio expertise (‘Are you an audio engineer, an acoustician, a proficient musician, or similar?’) and main language. Participants were screened so as to only include those aged 18 and over and with English as their main language. Demographic data is displayed in **Table 1**: as can be seen, participants for each study were broadly similar, with the exceptions that there were more participants aged 18–29 in the dog study, and fewer participants who self-identified as being audio experts in the engine study. These two features are addressed in the discussion section.

**Table 1:** Demographic data of participants for all studies.

		Soundscape	Nature	Manmade	Dogs	Engines
Age	18–29	41	35	35	70	33
	30–39	37	41	38	12	49
	40–49	18	11	13	14	10
	50–59	2	16	10	4	6
	60–69	2	2	2	0	2
	70–79	0	2	2	0	0
Sex	Male	39	36	63	46	43
	Female	61	61	38	54	57
	Rather not say	0	2	0	0	0
Audio expert	Yes	16	16	15	18	2
	No	84	84	85	82	98

*All values are percentages rounded up to the nearest whole percent.*

All stimuli (see Supplementary Table 1) were taken from Freesound^2^. Some were sourced directly from Freesound, others were sourced from ESC-50, a database of audio clips collected from Freesound and curated into categories by Piczak (2015). Where audio clips were sourced directly from Freesound, they were identified by searching filenames and descriptions using keywords corresponding to the sound names. Search results were sorted by number of downloads. Audio clips of synthesized sounds were rejected. Files were selected based upon subjective audio quality and duration: preference was given to clips that were ≤5 s, but where necessary clips were manually edited in duration. All stimuli were normalized to maximum amplitude of 3 dB below full-scale.

#### Top Level: Soundscape

*N* = 50 participants completed the initial study. Sixty stimuli (Supplementary Table 1) were selected so as to be representative of sounds described in a number of studies from the soundscape literature (Kawai et al., 2004; Gygi et al., 2007; Brown et al., 2011; Yang and Kang, 2013; Salamon et al., 2014). Brown et al. (2011) in particular place an emphasis on sounds occurring in multiple environmental contexts. Therefore an effort was made to include examples of sounds recorded indoors and outdoors where this was possible. In some cases these were recordings of sounds occurring outside, recorded from indoors, e.g., ‘Fireworks_2.’ In other cases these were recordings which were audibly recorded in different sized spaces, e.g., ‘Laughter_1’ sounded like it was recorded in a large room due to the audible reverberation, whereas ‘Laughter_2’ did not contain audible reverberation. All stimuli had duration of ≤5s.

#### Middle Level: Nature and Manmade Sounds

Analysis of top level sounds generated three principal categories, people, nature, and manmade. Of these, nature and manmade were considered the most interesting to explore further, since classification of the vocal and music sounds of the people category have been well studied previously (e.g., Pachet and Cazaly, 2000; Ververidis et al., 2004; Li and Ogihara, 2005; Giordano et al., 2010).

Each of the nature and manmade sound studies consisted of five exemplars of 13 sounds. All stimuli had duration of ≤5s. *N* = 45 participants completed the nature study; *N* = 48 completed the manmade study.

#### Bottom Level: Dog and Engine Sounds

To investigate category formation for single sound sources, an individual sound from each of the nature and manmade categories was selected, dog and engine sounds, respectively. In the interests of ecological validity, dog sounds were not restricted to 5 s duration; rather, clips were selected so as to sound natural (mean = 5.8 s, *SD* = 3.0 s). In some cases this meant selecting a section that sounded like a complete dog bark from a longer clip. *N* = 50 participants completed the dog’s study, whilst *N* = 49 completed the engines study.

### Analysis

#### Contingency Table

For each experiment the data from each participant were initially collected as a contingency table of 1s and 0s, where rows corresponded to individual sounds and columns corresponded to category names and where a 1 indicated that a sound had been placed in a given category, before being collated into a sounds × 5*N* categories contingency table of data from all participants. Each contingency table was consolidated by summing data where category names were the same or synonymous. Category names were initially processed by removing white space; removing special characters; removing the words ‘sound’ and ‘sounds’; removing numbers; converting to lower-case; and correcting spelling. Category names were then stemmed (e.g., ‘natural’ and ‘nature’ were reduced to ‘natur-’) before restoring each stem to the most common pre-stemming version of that word (e.g., ‘nature’). Categories which had either the same name following this process, or which were identified as synonyms by Microsoft’s synonym checker were then summed. This resulted in a contingency table which contained numbers other than 1 and 0 (see Supplementary Table 2 for details of which data were summed this way). Hereafter category names are referred to as ‘descriptive words.’ Consolidating the contingency tables reduced the number of descriptive words for soundscape sounds from 250 to 94; from 225 to 75 for nature; from 240 to 78 for manmade; from 250 to 59 for dogs; and from 245 to 96 for engines. A Pearson’s Chi-squared test found a dependence between rows and columns for all resulting contingency tables, demonstrating a significant relationship between sounds and descriptive words: soundscape, χ^2^(5487) = 7813.5, *p* < 0.001; nature, χ^2^(4736) = 8227.4, *p* < 0.001; manmade, χ^2^(4928) = 8989.7, *p* < 0.001; dogs, χ^2^(2494) = 3977.3, *p* < 0.001; engines, χ^2^(3705) = 3915.0, *p* < 0.001.

#### Correspondence Analysis

Each consolidated contingency table was submitted to a correspondence analysis (CA; see Greenacre, 1984; Lê et al., 2008), a method similar to principal component analysis but suitable for categorical rather than continuous data, in order to identify the principal dimensions of the data. CA was performed using the FactoMineR package (Lê et al., 2008) in R V3.3.3. This step was used to denoise the data prior to clustering (Husson et al., 2010), and to extract the dimensions of the similarity data so that sounds and descriptive words could be plotted in the same space. Dimensions with eigenvalues greater than would be the case were the data random were retained. For example, the top level soundscape contingency table had 60 rows (sounds) and 94 columns (descriptive words). Therefore were the data random the expected eigenvalue for each dimension would be 1.7% in terms of rows [1/(60-1)] and 1.1% in terms of columns [1/(94-1)], so all dimensions with eigenvalues greater than 1.7% were retained. The number of dimensions retained during correspondence analysis of each contingency table and the variance explained is displayed in Supplementary Table 3.

#### Cluster Analysis and Category Naming

Agglomerative hierarchical cluster analysis of the dimensions resulting from CA was performed using Ward’s criterion (see Husson et al., 2010), using FactoMineR. Taxonomies were derived by ‘slicing’ the resulting dendrograms at different heights and giving each resulting cluster a category name according to the descriptive words that contributed to that cluster. For all taxonomies apart from the dog taxonomy slices were performed so as to create all possible clusters above the height of the dendrogram at which the ratio of between-cluster inertia to total inertia was 0.1. Between-cluster inertia describes the deviation of the center of gravity of all clusters from the overall center of gravity, and total inertia describes this value summed with within-cluster inertia, i.e., the deviation of individuals from the center of gravity of each cluster. This ratio becomes greater with slices at higher levels of the dendrogram and cluster members become less similar. At slices at lower levels of the dendrogram this value becomes smaller and cluster members become more similar. The value of 0.1 was selected to allow populating the taxonomy with enough labels so as to be meaningful without compromising the quality of the labeling. In the case of the dog taxonomy, a ratio of 0.15 was chosen for the same reason.

The contribution of each descriptive word to each cluster was assessed by comparing global frequency (the total number of times sounds were assigned to a descriptive word) to the internal frequency for a given cluster (the number of times sounds within a cluster were assigned to that descriptive word). Significance of over-representation of each descriptive word within each cluster was assessed using a hypergeometric distribution (see Lê et al., 2008). The hypergeometric distribution describes the number of times an event occurs in a fixed number of trials, where each trial changes the probability for each subsequent trial because there is no replacement. Since the total number of descriptive words and the total number of times a given descriptive word was used is known, the probability *p* of a given descriptive word being used to describe sounds within a given cluster can be calculated. To illustrate this, consider the descriptive words applied to the soundscape dendrogram sliced into three clusters. Descriptive words that were over-represented in the first cluster are displayed in **Table 2** (see Supplementary Tables 4–8 for descriptive words corresponding to other clusters and other sounds). ‘People’ is the most over-represented descriptive word in this cluster: the sounds in this cluster were assigned to this descriptive word 257 times, out of a total of 357 times that sounds were assigned to this word. This first category of the soundscape taxonomy was therefore named ‘people.’

**Table 2:** Descriptive words that were significantly over-represented in the first cluster of the soundscape categorization data.

Descriptive word	Internal Freq.	Global Freq.	*p*	*v*-test
People	257	357	<0.001	22.109
Music	63	121	<0.001	7.438
Vocal	16	16	<0.001	6.608
Entertainment	18	20	<0.001	6.352
Chatter	10	10	<0.001	5.060
Changes	9	10	<0.001	4.316
Harmony	9	10	<0.001	4.316
Social	9	11	<0.001	3.974
Alive	9	11	<0.001	3.974
Enjoying	8	12	0.002	3.096
Marine	7	10	0.003	2.993
Species	9	16	0.005	2.801
Pleasant	8	14	0.008	2.658
Events	6	9	0.009	2.606
Relaxing	5	8	0.029	2.184


*This cluster was given the category name ’people’.*

This method of objectively naming taxonomic categories was sufficient in the majority (31 out of 56) cases. However, in other cases it was necessary to subjectively choose a descriptive word that was significantly over-represented but ranked lower to avoid repetition of category names (see Supplementary Tables 3–7). For example, in constructing the manmade taxonomy, a category was created with the name ‘home’ within a higher-level category also named ‘home.’ In these instances a name was subjectively chosen from a descriptive word lower down the table that better represented the content of the category. In this example ‘daily life’ was chosen for the category within ‘home’ that contained subcategories named ‘toilet’ and ‘food.’

#### Category Formation

##### Multinomial logit regression of descriptive words

The main aim of this study was to explore differences in how categories were formed between and within each level of the taxonomy. In order to examine this, each of the descriptive words (pre-consolidation) used in each of the studies were independently coded by three people: the first author and two acoustics doctoral students. All three are native speakers of English. Words were coded as describing either the *source-event* (referring to the inferred source of the sound), the *acoustic* signal (explicitly referring to the sound itself), or a *subjective-state* (describing an emotional state caused by the sound or of the sound source). Word types were determined by agreement between at least two of the three coders: this criteria was met for all words (see Supplementary Table 9). Multinomial logit regression models were used to compare the likelihood of each type of descriptive word being used to describe sounds at each level of the taxonomy and for each group of sounds. In each case the dependent variable was the type of descriptive word used (e.g., subjective-state vs. source), and the independent variables were level of the taxonomy (e.g., top vs. middle) or the sound type (e.g., nature vs. manmade). Multinomial logit regression models produce log-odds coefficients (*B*) that can be expressed as an odds ratio (*e^B^*). These describe how many times more likely a type of descriptive word is used relative to another type of descriptive word, at a given level of the taxonomy relative to another level, or for a sound type relative to another sound type.

In order to assess the effect that providing labels for the tiles had on how categories were formed, a supplementary top level study was performed in which tiles were labeled with pseudorandomized numbers. Multinomial logit regression models demonstrated that providing text labels did not significantly change the proportion of word types used (see Supplementary Tables 10, 11).

##### Post hoc analysis

To explore strategies for categorization further, the arrangement of sounds and descriptive words within the space created by the dimensions elicited by CA were examined. Based upon the results of the multinomial logit regression models a *post hoc* decision was taken to explore arousal and valence for the descriptive words used for dog sounds. A correlation between the coordinates of words describing subjective-states and measures of valence and arousal for those words was calculated. The arousal and valence values for the words were taken from a scored dataset of 13915 lemmas (Warriner et al., 2013; see Supplementary Table 12).

Similarly, the multinomial logit regression models indicated further analysis of engine sounds should use acoustic features. This was based upon the finding that explicit assessment of the acoustic signal accounted for categorization. The coordinates of engine sounds and two simple acoustic features commonly used by industry to assess product sounds, fluctuation strength and sharpness, were tested for correlation. Fluctuation strength is a measure of amplitude modulation below 20 Hz, whilst sharpness is a measure of high-frequency content. Both measures account for the perceptual distance between frequencies by dividing the signal into critical bands using the Bark scale. Fluctuation strength is measured in units of vacil where 1 vacil is defined as the fluctuation strength produced by a 1000 Hz tone with a sound pressure level of 60 dB, 100% amplitude modulated at 4 Hz. Sharpness is measured in acum, where 1 acum has the equivalent sharpness of a narrow-band noise with a center frequency of 1000 Hz, a bandwidth of 1 critical band, and a sound pressure level of 60 dB. Both fluctuation strength and sharpness were evaluated with dBFA software using the criteria of Zwicker and Fastl (2013). Since the presentation level of the stimuli in this study was not controlled due to participants being recruited online, both sharpness and fluctuation strength calculations were referenced to a 1000 Hz sine wave with an amplitude of 1 Pa, which equates to a sound pressure level of 71 dB at full-scale. Note that of interest here is the relative rather than absolute fluctuation strength and sharpness.

Association between the coordinates of descriptive words (dogs) and sounds (engines), respectively, are reported using one-tailed Spearman’s Rho (*r_s_*) and Pearson’s product-moment (*r*) correlations. No attempt was made to identify acoustic correlates of the dimensions of other data, since categorization in these cases was accounted for by other cues.

## Results

### Category Formation

The main purpose of the current study was to explore differences in the way that sound categories are formed between and within different levels of category hierarchy. The types of words used to describe sounds at each level of the taxonomy and for each type of sound are presented in **Table 3**. A series of multinomial logit regression models were fitted to the descriptive word data (see **Table 4**). The likelihood of using words describing source-event, signal, and subjective-states did not significantly differ between the middle and top levels of the taxonomy: at these levels the majority of words used described source-events (top, 81%; middle, 75%). However, there were significant differences between bottom and top, and bottom and middle levels. Expressed as an odds ratio (*e^B^*), there were 11 times the odds of using a word that described a subjective-state rather than the source-event at the bottom level compared to the top level, and 4.7 times the odds of using a word describing the acoustic signal rather than the source-event. On the other hand, there were 0.4 times the odds of using a word describing the acoustic signal rather than a subjective-state at the bottom level compared to the top level.

**Table 3:** Percentages of different types of descriptive words used at each level of the taxonomy and for each type of sound.

	Top	Middle		Bottom	
Source	81.2	75.1		42.0	
Acoustic	14.8	17.7		35.6	
Subjective	4.0	7.2		22.4	
	**Soundscape**	**Nature**	**Manmade**	**Dogs**	**Engines**
Source	81.2	75.6	74.6	24.0	60.0
Acoustic	14.8	13.8	21.7	34.0	37.1
Subjective	4.0	10.7	3.8	42.0	2.9


**Table 4:** Results of the multinomial logit regression models.

		*B*	*e^B^*	*SE*	*p*
Middle vs. Top	Subjective vs. Source	0.65	1.9	0.37	0.08
	Acoustic vs. Source	0.26	1.30	0.22	0.22
	Acoustic vs. Subjective	-0.39	0.68	0.41	0.35
Bottom vs. Top	Subjective vs. Source	2.40	11.0	0.34	<0.001^∗^
	Acoustic vs. Source	1.54	4.7	0.21	<0.001^∗^
	Acoustic vs. Subjective	-0.86	0.4	0.38	0.022^∗^
Bottom vs. Middle	Subjective vs. Source	1.74	5.7	0.22	<0.001^∗^
	Acoustic vs. Source	1.27	3.6	0.16	<0.001^∗^
	Acoustic vs. Subjective	-0.47	0.6	0.24	0.049^∗^
Nature vs. Manmade	Subjective vs. Source	1.03	2.8	0.41	0.011^∗^
	Acoustic vs. Source	-0.47	0.6	0.25	0.063
	Acoustic vs. Subjective	-1.50	0.2	0.45	<0.001^∗^
Dogs vs. Engines	Subjective vs. Source	3.60	36.7	0.42	<0.001^∗^
	Acoustic vs. Source	0.83	2.3	0.22	<0.001^∗^
	Acoustic vs. Subjective	-2.78	0.1	0.42	<0.001^∗^


*In each case the dependent variable was the type of descriptive word used (e.g., subjective-state vs. source), and the independent variables were level of the taxonomy (e.g.*, *top), or the sound type (e.g.*, *nature).*

There were also 5.7 times the odds of using a word describing a subjective-state rather than the source-event at the bottom level compared to the middle level, and 3.6 times the odds of using a word describing the acoustic signal rather the source-event. On the other hand there were 0.6 times the odds of using a word describing the acoustic signal rather than a subjective state at the bottom level compared to the middle level.

Within the middle level there were 2.8 times the odds of using a word that described a subjective-state rather than the source-event when describing nature sounds compared to manmade sounds. However, there were only 0.2 times the odds of using a word describing the acoustic signal rather than a subjective-state. Within the bottom level there was 36.7 times the odds of using a word that described a subjective-state rather than the source-event when describing dog sounds compared to engine sounds, and 2.3 the odds of using a word describing the acoustic signal rather than the source-event. However, there were only 0.1 times the odds of using a word describing the acoustic signal rather than a subjective-state when describing dog sounds compared to engine sounds.

#### Top and Middle Level Sounds: Soundscape, Nature, and Manmade

**FIGURE 1:**
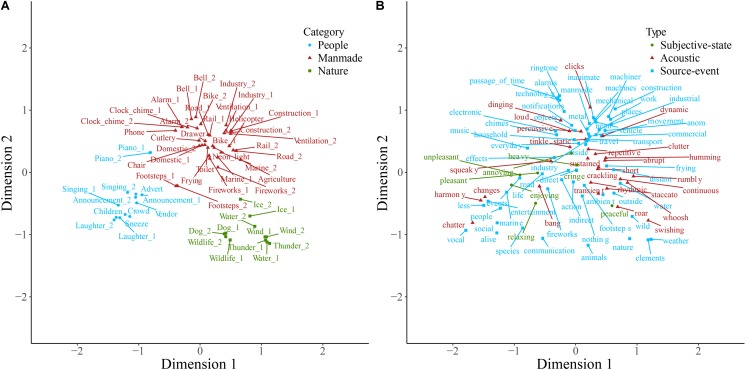
Soundscape sounds **(A)** and descriptive words **(B)** plotted on the first two dimensions of categorization data. Note that the dimensions are the same in both panels. Sounds **(A)** are colored according to which of the main categories they belong to, and descriptive words **(B)** are colored according to type. Labels are displaced from their corresponding data point, indicated by a connecting line, to avoid overlapping.

**FIGURE 2:**
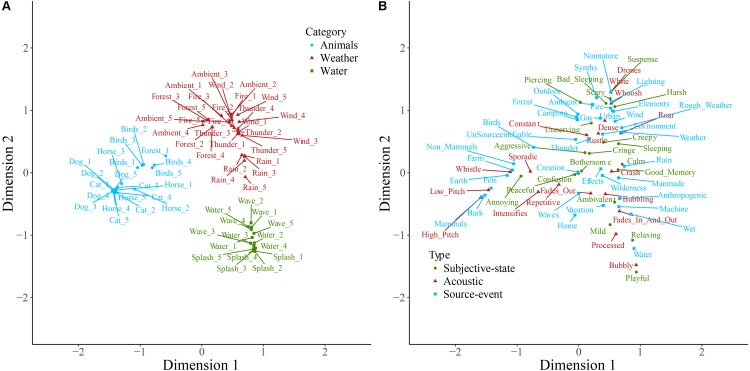
Nature sounds **(A)** and descriptive words **(B)** plotted on the first two dimensions of categorization data. Note that the dimensions are the same in both panels. Sounds **(A)** are colored according to which of the main categories they belong to, and descriptive words **(B)** are colored according to type. Labels are displaced from their corresponding data point, indicated by a connecting line, to avoid overlapping.

**FIGURE 3:**
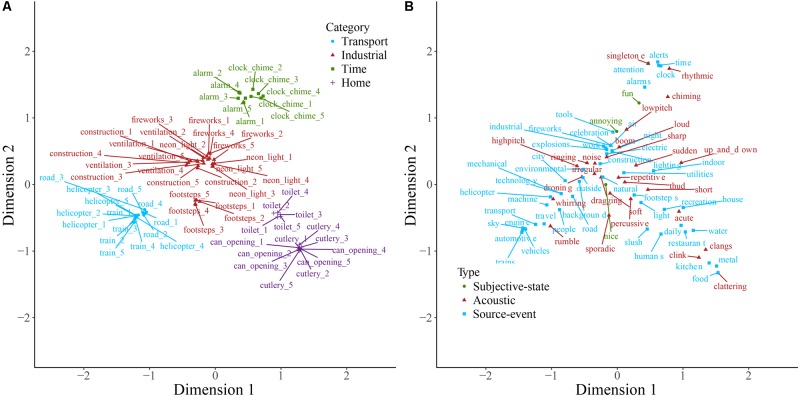
Manmade sounds **(A)** and descriptive words **(B)** plotted on the first two dimensions of categorization data. Note that the dimensions are the same in both panels. Sounds **(A)** are colored according to which of the main categories they belong to, and descriptive words **(B)** are colored according to type. Labels are displaced from their corresponding data point, indicated by a connecting line, to avoid overlapping.

Sounds and descriptive words for soundscape, nature, and manmade sounds are plotted on the first two dimensions resulting from correspondence analysis of each of the contingency tables in **Figures 1–3**. Note that the dimensions are the same in both panels of each plot. Note also that here and in other two-dimensional plots the descriptive words are those retained following consolidation of the contingency table, and therefore the ratio of descriptive term types differs from that described above. Some insight into category formation is gained by inspecting sounds at the boundaries of the categories. In the top-level study in **Figure 1**, sounds such as footsteps and cutlery are categorized as manmade, though they are closer to the people category than manmade sounds like helicopter and ventilation. This suggests that at the top level, category formation is based upon identification of the sound source-event. Similarly, as might be expected, the rain sounds in **Figure 2** are, despite being part of the weather category, close in space to the water category. In **Figure 3**, the footstep sounds are closer to the home and transport categories than are other industrial sounds. That footsteps are categorized as industrial within the manmade taxonomy, and as manmade within the soundscape taxonomy, suggests that in this instance the sounds were categorized by their acoustic features (e.g., impacts) rather than by sound source-event *per se*.

#### Bottom Level

##### Dogs

**FIGURE 4:**
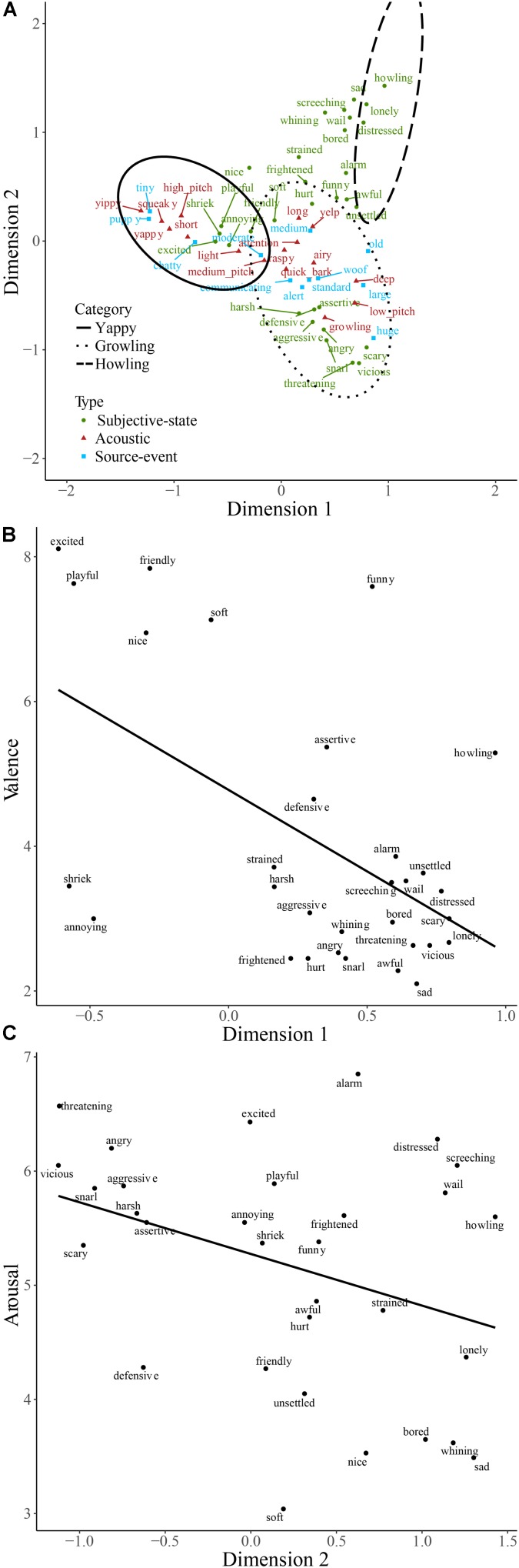
Descriptive words used to describe dog sounds plotted on the first two dimensions of categorization data **(A)**. Words are colored according to type. The regions of the two-dimensional space corresponding to the three main categories of dog sounds are indicated by solid, dashed, and dotted lines. Valence **(B)** and arousal scores **(C)** of affect-judgments are plotted against their coordinates on dimensions 1 and 2, respectively.

The majority of words used to describe dog sounds were those describing subjective-states (**Table 3**), and the odds of using this type of word rather than words describing source-event or acoustic signal was far greater than for engine sounds (**Table 4**). In order to explore this further, dog sounds and descriptive words are plotted on the first two dimensions resulting from correspondence analysis of the contingency table in **Figure 4A**. The first two dimensions accounted for 50.5% of the total variance. The space populated by the howling category contains descriptive words, such as ‘sad,’ ‘lonely,’ and ‘distressed’; that populated by the yappy category contains descriptive words, such as ‘puppy,’ ‘squeaky,’ and ‘excited’; and the space populated by the growling category contains descriptive words, such as ‘aggressive,’ ‘snarling,’ and ‘scary.’ More generally, the descriptive words change from being broadly positive to broadly negative along the first dimension, and from describing states of higher to lower arousal along the second dimension. The coordinates of subjective-states on the first dimension were found to correlate with valence scores (**Figure 4B**; *r_s_*(29) = -0.53, *p* < 0.001), and their coordinates on the second dimension were found to correlate with arousal scores (**Figure 4C**; *r_s_*(29) = -0.35, *p* = 0.03). This is consistent with participants using subjective-states corresponding to valence and arousal to differentiate the dog sounds.

##### Engines

**FIGURE 5:**
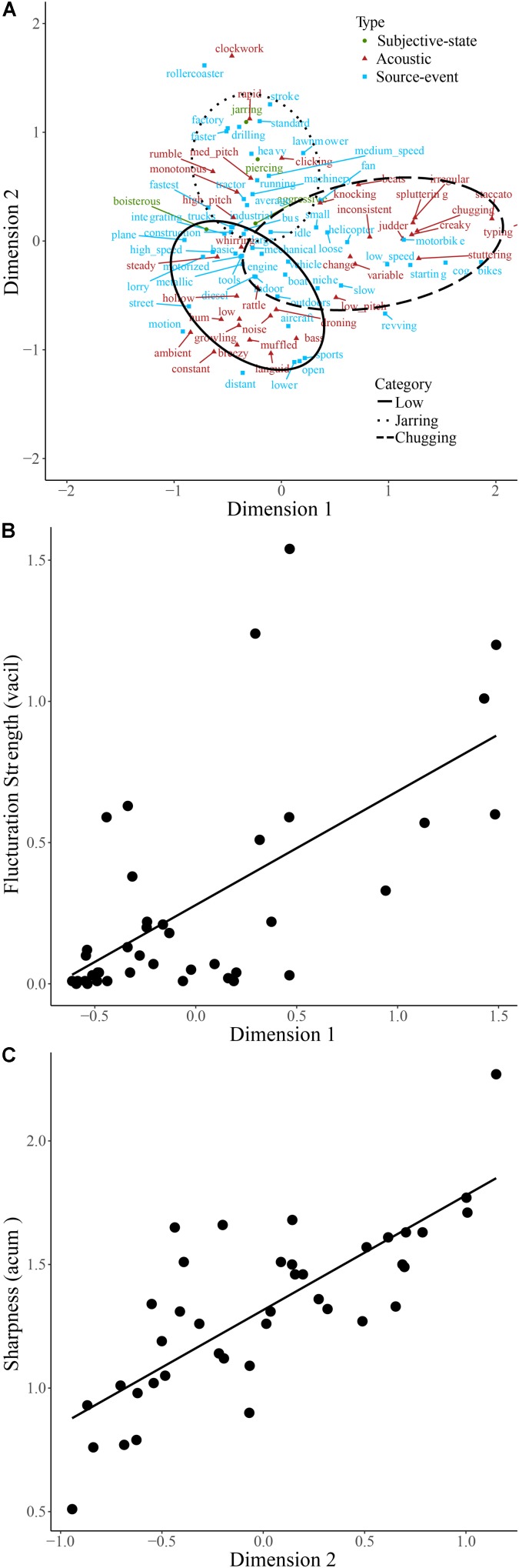
Descriptive words used to describe engine sounds plotted on the first two dimensions of categorization data **(A)**. Words are colored according to type. The regions of the two-dimensional space corresponding to the three main categories of engine sounds are indicated by solid, dashed, and dotted lines. Fluctuation strength **(B)** and sharpness **(C)** of engine sounds are plotted against their coordinates on dimensions 1 and 2, respectively.

Engine sounds and descriptive words are plotted on the first two dimensions of the categorization data, accounting for 33.8% of the total variance, in **Figure 5A**. The chugging category is located on the positive half of dimension 1 and at approximately 0 on dimension 2. The low and jarring categories cover areas from approximately -1 to +0.5 on dimension 1, located below and above 0 on dimension 2, respectively. Since words describing subjective-states made up just 2.5% of descriptive words, category formation of engine sounds differs from dog sounds. Compared to dog sounds the odds of using words explicitly describing the acoustic signal rather than a subjective-state were significantly greater for engine sounds. Visual inspection of **Figure 5A** shows that words relating to temporal regularity (e.g., ‘constant,’ ‘steady,’ and ‘rumble’) are located to the left of the plot and that those relating to temporal irregularity (e.g., ‘staccato,’ ‘stuttering,’ and ‘chugging’) are located to the right. This suggests that the first dimension relates to the fluctuation of the sound. Likewise, dimension 2 of **Figure 5A** may relate to the sharpness of the sound, with terms, such as ‘jarring,’ ‘drilling,’ and ‘piercing’ located toward the top of the plot and terms, such as ‘languid,’ ‘muffled,’ and ‘hum’ toward the bottom. Consistent with these being the basis for category formation of engine sounds, fluctuation strength and sharpness of the engine sounds were found to correlate with the coordinate of each sound on dimension 1 (**Figure 5B**; *r_s_*(38) = 0.81, *p* < 0.001) and dimension 2 (**Figure 5C**; *r*(38) = 0.83, *p* < 0.001), respectively.

### Taxonomies

**FIGURE 6:**
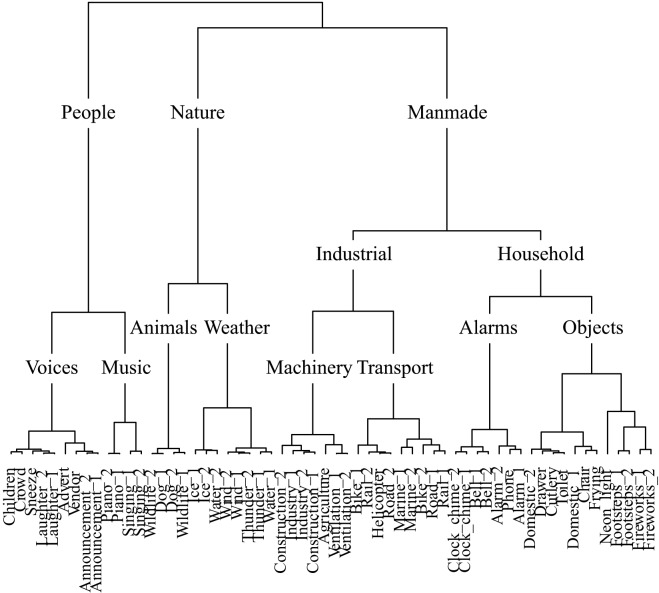
The soundscape taxonomy generated by hierarchical cluster analysis of the principal dimensions resulting from correspondence analysis.

**Figure 6** displays the taxonomy derived from cluster analysis of the dimensions of the soundscape contingency table. Sounds are initially partitioned into three categories: people, nature, and manmade. Note that **Figure 6** is limited in depth by the number of sounds used in the top level soundscape experiment (60). Thus the music category, for example, contains only piano and singing sounds. However, the depth of any branch could be expanded by applying the same experimental method to a restricted set of sounds; for example, to 60 different music sounds.

**FIGURE 7:**
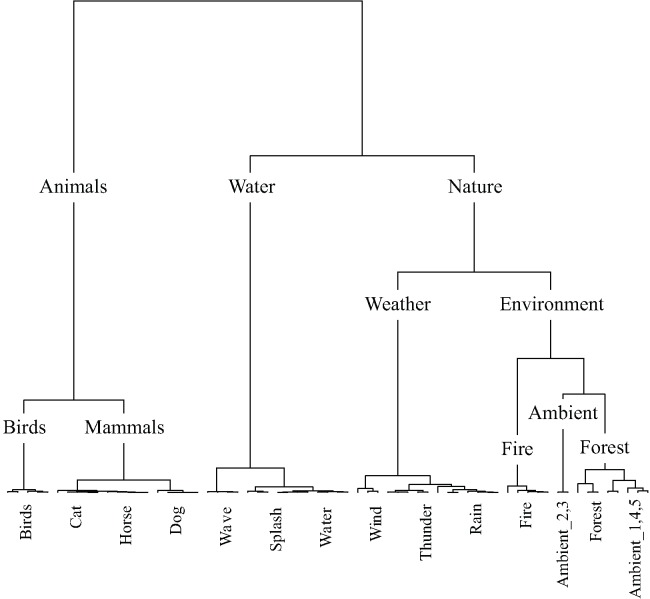
The nature taxonomy generated by hierarchical cluster analysis of the principal dimensions resulting from correspondence analysis. Note that five exemplars of each sound were used in this study. All five exemplars of each sound were categorized together, except for the case of ambient where two exemplars were categorized together in a category by the same name and three were categorized as forest sounds.

**FIGURE 8:**
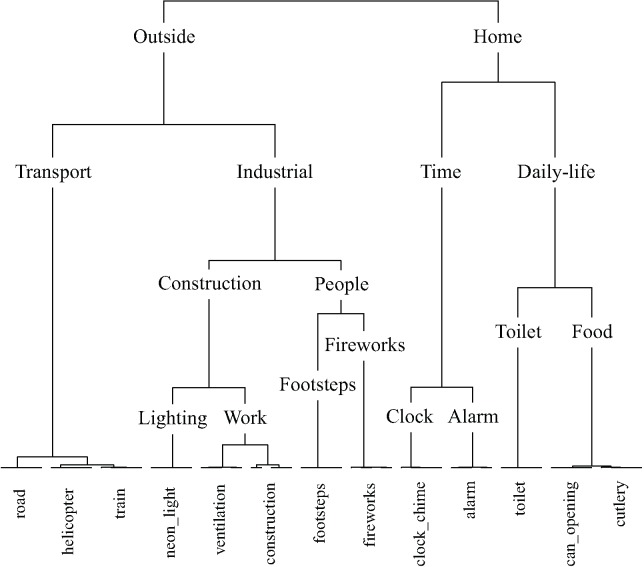
The manmade taxonomy generated by hierarchical cluster analysis of the principal dimensions resulting from correspondence analysis. Note that five exemplars of each sound were used in this study. In all cases all five exemplars of each sound were categorized together.

**Figure 7** displays the taxonomy derived from cluster analysis of the dimensions of the nature contingency table. The three main categories are animals, water, and nature. **Figure 8** displays the taxonomy derived from cluster analysis of the dimensions of the manmade contingency table. The first division is between outside and home sounds. Outside sounds consist of two categories, transport and industrial. The home category divides into time and daily-life.

**FIGURE 9:**
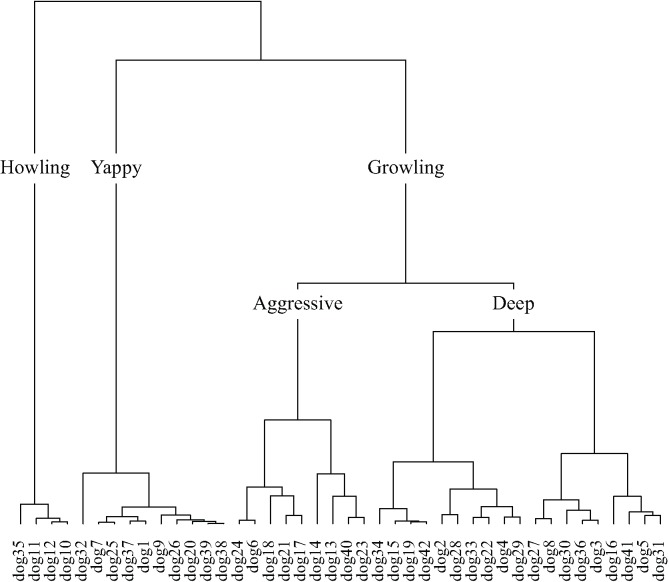
The dog taxonomy generated by hierarchical cluster analysis of the principal dimensions resulting from correspondence analysis.

**FIGURE 10:**
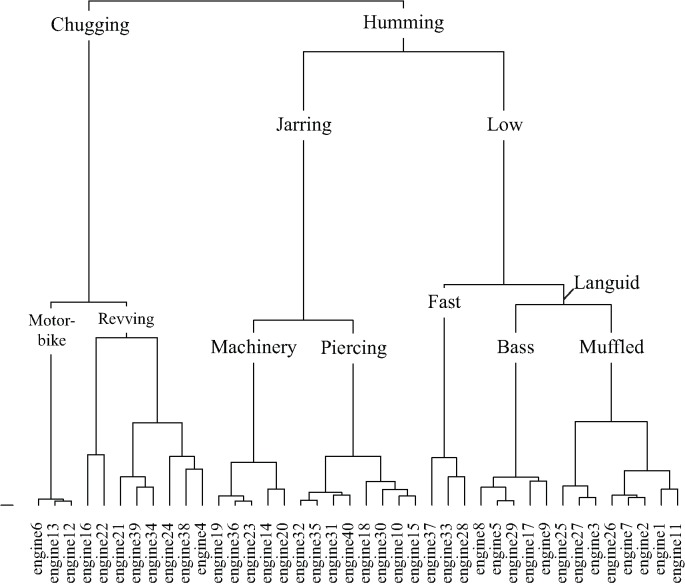
The engine taxonomy generated by hierarchical cluster analysis of the principal dimensions resulting from correspondence analysis.

Taxonomies derived from cluster analysis of the dimensions of the dogs and engines contingency tables are displayed in **Figures 9**, **10**, respectively. Dog sounds are initially partitioned into howling, yappy, and growling. Engine sounds are initially partitioned into chugging and humming. Chugging sounds are further divided into motor-bike and revving sounds; humming sounds are further divided into jarring and low sounds.

## Discussion

### Category Formation

The main aim of the study was to use verbal correlates of sound categorization to explore differences between how categories are formed between and within different levels of category hierarchy. The results demonstrate a significant difference between the types of words used to describe categories of sounds, between the bottom and top levels, and between the bottom and middle levels of the emergent taxonomy. The findings are consistent with source-event identification being the principal cue for category formation at the top and middle levels of the taxonomy. This agrees with previous suggestions that at this level of differentiation, sounds are typically categorized by perceived similarities between sound sources rather than by abstracted acoustic features *per se* (e.g., Gaver, 1993; Marcell et al., 2000; Dubois et al., 2006; Guastavino, 2006; Houix et al., 2012). It also concurs with everyday listening being primarily concerned with gathering information about sound sources (Schubert, 1975; Gaver, 1993). However, despite evidence for source-event identification being the principal cue by which categories were formed within the middle level, it was found that nature sounds were more likely than manmade sounds to be described by a subjective-state compared to a source-event, and less likely to be described by explicit reference to the acoustic signal compared to a subjective-state. When categorizing multiple examples of a specific sound source from the nature category (dogs), participants were even more likely to use words describing a subjective-state compared to a source-event, relative to when categorizing multiple examples of a specific sound-source from the manmade category (engines), and even less likely to use words describing the acoustic signal compared to a subjective-state.

Taken together, these results provide strong evidence that the use of cues for forming categories differs both between and within levels of hierarchy. It is likely that in the case of dog sounds subjective-states represent the greatest potential for differentiating sounds, whereas for engine sounds this strategy is insufficient or meaningless, and a strategy based upon explicit assessment of the acoustic properties of the sounds is employed.

#### Categorization Based Upon Explicit Judgment of the Acoustic Signal

In the case of engine sounds, although the amount of variance explained by two dimensions was low (relative to, e.g., Kawai et al., 2004; Gygi et al., 2007; Axelsson et al., 2010; Hong and Jeon, 2015) they strongly correlated with fluctuation strength and sharpness, respectively, suggesting that these acoustic features were used to differentiate and categorize these sounds. It is notable that despite these acoustic properties being regularly used in product sound evaluation within the automotive industry (e.g., Nor et al., 2008; Wang et al., 2014) to the authors’ knowledge this is the first time that a *spontaneous* strategy for differentiating engine sounds using sharpness and fluctuation strength cues has been demonstrated, providing ecological validity to these measures.

One important feature of the approach taken in the present study is that it is possible to interpret the perceptual correlates of fluctuation strength and sharpness of engine sounds using the spontaneously generated descriptive words. For example, as fluctuation strength increases the engine sounds become more ‘chugging’ and ‘judder’-like etc. This is to say, the data represents a mapping between these acoustic features and their subjective meaning in relation to engine sounds.

#### Categorization Based Upon Valence and Arousal

The circumplex model of affect regards valence and arousal as being ‘core affect’ (Russell, 1980, 2003; Posner et al., 2005) and emotions as being the perceived potential for a stimulus to cause a change in this core affect. Rather than having discrete borders, emotions are understood as being instantiated out of the subjective interpretation of patterns of neurophysiological activity in the mesolimbic system and the reticular formation, responsible for the sensations of valence and arousal, respectively. Previous work has employed the concept of core affect as, for example, an organizing principle for musical sounds (e.g., Gomez-Marin et al., 2016), and as the basis for automatic classification of sounds (Fan et al., 2016). Our finding of an association between the first two dimensions of the dog categorization data and valence and arousal lends support to the circumplex model of affect. It appears to be a meaningful framework for understanding human categorization of *some* sound types.

Whilst Bergman et al. (2016) found that valence and arousal ratings together mapped onto a dimension of dissimilarity data of everyday sounds explicitly chosen so as to produce an emotional response, we have shown that valence and arousal independently correspond to the first two dimensions of the data from a task where participants were free to categorize by whichever cues they chose. An interesting feature of the method presented here is in the potential for using the spontaneously generated descriptive words that are mapped onto dimensions corresponding to valence and arousal to interpret the perception of affective qualities within the context of dog sounds. For example, it can be said that dog sounds that cause a large valence response are those that are perceived as ‘excited,’ ‘playful,’ and ‘friendly’ (**Figure 4B**), and those that cause a large arousal response are those perceived as ‘vicious,’ ‘snarling,’ and ‘threatening’ (**Figure 4C**).

### Taxonomies

The present study has produced five sound taxonomies using a method where participants were free to use whichever cues they prefer to form categories: a ‘top level’ soundscape taxonomy, ‘middle level’ nature and man-made sounds taxonomies, and ‘bottom level’ dog and engine sounds taxonomies. Previous attempts to taxonomize environmental sounds have taken a variety of approaches (e.g., Gaver, 1993; Gygi et al., 2007; Brown et al., 2011; Lemaitre and Heller, 2013; Salamon et al., 2014; Lindborg, 2015). The framework for standardized reporting of events within a soundscape based upon expert opinion produced by Brown et al. (2011) has proven particularly influential in soundscape research, although strictly speaking it is not a taxonomy *per se*. The taxonomies we presented here improve on previous accounts because they are generated experimentally using statistical modeling, being based on the responses of the general public.

The soundscape presented by Brown et al. (2011) is initially divided into indoor and outdoor sounds, with sounds within both further divided into urban, rural, wilderness, and underwater environments. Sounds are then categorized by sound source the same way within each environment. Of the taxonomies presented here, the manmade taxonomy is the only one to have a principal division between environmental contexts, outside and home; for these sounds the environment with which they are most commonly associated was a strong organizing principle. The soundscape taxonomy presented here does not have the same initial division by environment; rather, sounds are categorized by source-event. It is note-worthy that the categories of sounds prescribed by Schafer (1993), based upon a review of descriptions of sounds in literature, anthropological reports, and historical documents, bear resemblance to a number of categories to have spontaneously emerged here. Schafer’s categories: natural, human, society, mechanical, and indicators, are similar to the categories in the soundscape taxonomy: nature, people, manmade, machinery, and alarms, respectively.

It is interesting to note which of the sounds that were used in both the top level soundscape study and the middle level manmade study were categorized differently: the neon-light sound was categorized as ‘manmade – household – objects’ within the soundscape taxonomy, but as ‘outside – industrial – construction’ within the manmade taxonomy. Both footstep sounds and fireworks sounds were categorized as ‘manmade – household – objects’ within the soundscape taxonomy, but as ‘outside – industrial – people’ within the manmade taxonomy. This is likely to be due in part to an effect of context; within the context of the set of sounds used in the soundscape study the impact sound of footsteps, the snapping and cracking sound of the fireworks, and the popping sound of the neon-light led to these being deemed as belonging together in an objects category with other sounds with similar acoustic properties such as the sound of rattling cutlery. However, within the context of the sounds used in the manmade study the meaning of the cutlery sounds was more strongly associated with the sound of a can opening, whilst the neon-light sound was more strongly associated with industrial sounds and the footsteps and fireworks sounds were grouped together in a separate people category. Notably, the people category of the manmade taxonomy is somewhat out of place, due to the meaning of the footstep and firework sounds arguably being least similar to the other sounds used in the manmade study, and the least clearly manmade.

### Methodology Considerations and Implications for Soundscape Research

Contrary to the results presented here, a number of soundscape studies have reported principal dimensions relating to subjective-states. This may be due in part to the use of prescriptive semantic differentials ratings in previous studies. Cain et al. (2013) assessed the perception of soundscapes in their entirety and found the principal dimensions ‘calmness’ and ‘vibrancy,’ but specifically asked people to rate soundscapes for their ‘calmness,’ ‘comfort,’ how fun they were, how confusing they were, and how intrusive they were. Yu et al. (2016) found a principal dimension ‘preference,’ but used semantic differential scales containing terms, such as ‘beautiful,’ ‘relaxing,’ and ‘comfortable.’ Kang and Zhang (2010) reported ‘relaxation’ as the first dimension, using semantic differential scales, such as ‘agitating,’ ‘comfort,’ ‘pleasant,’ and ‘quiet.’ Similarly, in work more comparable to the present study, Payne et al. (2007) assessed the perception of individual sounds heard within the soundscape and found a dimension ‘pleasantness,’ but again explicitly used the semantic scales ‘pleasantness’ and ‘stressful.’ Unlike the studies mentioned above, Kawai et al. (2004) inferred ‘preference’ and ‘activity’ as the two principal dimensions resulting from PCA based upon a semantic differential task using terms generated by participants to describe the sound groupings. However, these were not the original, spontaneously generated names given to groups of sounds, which described the identified sound sources (‘sounds of nature,’ ‘sounds of water’), rather participants were then instructed to further describe sounds within the group with a word that ‘best represented the overall representation’ and a word with the opposite meaning, in order to construct semantic differential scales. It is likely that this instruction to provide opposing descriptors biased the participants to produce adjectives rather than sound sources.

While these previous studies demonstrate that it is possible to differentiate soundscapes and the quotidian sounds in terms similar to valence and arousal when instructed to do so, our study indicates that this strategy is unlikely to be used spontaneously. Valence and arousal does not reflect the cognitive processes used in sound categorization for four of the taxonomies. This is consistent with Osgood (1969). He noted that findings generated by his own EPA framework may be a phenomenon that only occurs with forced use of adjectives, e.g., he notes that the concept ‘tornado’ is regularly rated as highly ‘unfair,’ despite this making no literal sense.

As noted previously, there was a significant difference in participant age between the dogs and engines studies. It cannot therefore be ruled out that the differences in categorization strategy were due to the larger proportion of 18–29 year olds that took part in the dogs study employing a strategy based upon subjective-states. However, it is suggested that the effect is much more likely to have been caused by the availability and utility of the strategies, reflecting the difference between an animate object with agency and an inanimate machine. In the case of dog sounds, the range and perceived magnitude of affective qualities meant that categorization was easiest based upon this measure. In the case of engine sounds affective qualities were less distinct, whereas the meaning could be better described by the acoustic signals themselves. It is also notable that the strategy used in the engines study was one based upon explicit judgments about the acoustic properties of the sounds despite the participants of this study having the smallest proportion of audio experts.

The taxonomies presented here represent the meanings attributed to the sounds in each study. This differs conceptually to the taxonomy presented by Brown et al. (2011), which was presented as a framework for standardizing soundscape reporting, and so tried to account for as many combinations of source and context as possible. It is noted that differences between the two taxonomies might reflect differences in the sounds selected. Take for example the distinction between ‘nature – wildlife’ and ‘domestic animals’ in Brown et al. (2011). This was not found in our study, although maybe a greater sample of both domestic and wild animal sounds would have changed this finding. More generally, the categories presented here are not intended to be taken as absolute. Although the sounds used were chosen to represent sounds frequently reported in the soundscape literature, it must be acknowledged that a different selection of sounds could have resulted in different categories emerging. Context is doubtless an important component of sound perception; for example, one’s activity within the context of the soundscape is likely to affect the way in which individual sounds are evaluated (Cain et al., 2008). The procedure described here did not account for such contextual factors; rather the presented taxonomies reflect categories of detached sounds. Similarly, it is likely that in real-world situations perception of the soundscape is shaped by interactions between acoustic and visual cues (e.g., Ge and Hokao, 2005).

## Conclusion

Taxonomies of sounds commonly found in soundscape studies, nature sounds, manmade sounds, dog sounds, and engine sounds are presented. Statistical analysis of the frequency with which types of descriptive terms were used demonstrate that whilst participants primarily categorized soundscape, nature, and manmade sounds based upon sound source-event, two further strategies were used to categorize dog and engine sounds based upon subjective-states and explicit assessment of the acoustic signal, respectively. The dimensions of the dog categorization data corresponded to valence and arousal scores. The dimensions of the engine categorization data corresponded to descriptive terms relating to fluctuation strength and sharpness, and were found to correlate with these two acoustic features. The method used here allows for the interpretation of the subjective meaning of these features within the context of engine sounds: fluctuation strength was perceived as ‘chugging’ and ‘stuttering,’ whilst sharpness was perceived as ‘jarring’ and ‘piercing.’ Similarly, it can be said that valence is perceived as ‘yappy’ and ‘excited’ within the context of dog sounds, and arousal as ‘aggressive’ and ‘growling.’ The results of the present study suggest that careful consideration should be given to the appropriateness of the use of prescriptive semantic differential methods in future work.

## Ethics Statement

This study was carried out in accordance with the recommendations of UK RIO Code of Practice for Research (2009) and the University of Salford Research, Innovation and Academic Engagement Ethical Approval Panel, with written informed consent from all subjects. All subjects gave written informed consent in accordance with the Declaration of Helsinki. The protocol was approved by the University of Salford Research, Innovation and Academic Engagement Ethical Approval Panel.

## Author Contributions

OB designed the study, collected and analyzed the data, and drafted the manuscript. TC contributed to the design of the study, interpretation of the data, and hosted and implemented the web platform. WD contributed to the design of the study and interpretation of the data.

## Conflict of Interest Statement

The authors declare that the research was conducted in the absence of any commercial or financial relationships that could be construed as a potential conflict of interest.

## Footnotes

1 http://www.sound101.org

2 www.freesound.org
